# Skin of Color Dermatology Representation in American College of Mohs Surgery Educational Cases on Instagram: Content Analysis

**DOI:** 10.2196/44103

**Published:** 2023-01-13

**Authors:** Morgan Zueger, Paige Nahod, Nathaniel A Marroquin, Mindy D Szeto, Hamza Ajmal, Olnita Martini, Colin Burnette, Alyssa P Quinn, Garrett Furth, Michelle Militello, Robert P Dellavalle

**Affiliations:** 1 College of Osteopathic Medicine Rocky Vista University Greenwood Village, CO United States; 2 Department of Dermatology University of Colorado Anschutz Medical Campus Aurora, CO United States; 3 College of Osteopathic Medicine Nova Southeastern University Davie, FL United States; 4 Department of Dermatology HCA Florida Orange Park Hospital Orange Park, FL United States; 5 Dermatology Service US Department of Veterans Affairs Rocky Mountain Regional VA Medical Center Aurora, CO United States

**Keywords:** skin of color, inequality, color, skin, social media, content analysis, dermatology, cancer, diversity, equity, inclusion, representation, Mohs surgery, skin tone, dermatologic surgery, Instagram, education, medical education

Social media is a prominent avenue for health care information delivery. The American College of Mohs Surgery (ACMS) in particular is an established professional organization for dermatologic surgeons, and its most popular social media platform is @mohs.college on Instagram (2000+ followers). As a respected resource for Mohs surgery, the ACMS and @mohs.college provides education for patients, students, and dermatologic surgeons.

While social media can be highly educational, skin cancers in skin of color (SoC) patients are often underdiagnosed or diagnosed at later stages with worse outcomes [1], likely due in part to inadequate training and exposure to the visual appearance of conditions on different skin tones. Thus, we assessed SoC representation in the popular weekly “Flap Friday” content on the @mohs.college page, featuring pre- and postprocedure Mohs cases (Figure 1). Two independent raters categorized and tabulated patients’ constitutive skin tones (light, fair, medium, or dark) following previously published methods [2], with discrepancies resolved by independent tiebreakers and consensus meetings. While Fitzpatrick phototypes are commonly used, the scale is intended to define sun sensitivity and reactivity rather than pigmentary phenotypic appearance. White skin phenotypes may be predictive of Fitzpatrick classification, while nonwhite phenotypes may not [2]. Therefore, this 4-tone scale was used to categorize photos, especially since the patient’s sun reactivity may not be known.

Out of 114 weeks (July 2020 to September 2022), 93 “Flap Friday” cases were analyzed. Overall, 83.9% (78/93) were considered to be of light skin tones, and 16.1% (15/93) were considered fair. Interrater agreement was 77.4%, and reliability was substantial with a Cohen κ of 0.643. None of the cases depicted medium or dark skin tones, although the proportions of fair (darker) skin tones were observed to increase every year from 14.3% (3/21) in 2020 to 25.9% (7/27) in 2022 (Figure 2).

These results corroborate current trends [3] where only up to 15% to 18% of resources included SoC patients. A recent analysis of 2451 cases in *JAAD Case Reports* revealed that for cases published in 2015, pictured skin tones were perceived as 73% light, 15% medium, and 12% dark; promisingly, percentages of SoC increased slightly in later years [4]. Furthermore, from 1995 to 2010, it was seen that African American patients received Mohs surgery in 44.2% of skin cancer visits, compared to 9.6% for Caucasians [5]. Given this high Mohs utilization and SoC skin cancer underdiagnosis, academic resources, including social media from prominent national organizations such as the ACMS, should be encouraged to increase SoC exposure and alleviate SoC representation gaps to improve care for the United States’s increasingly diverse population. Parity in social media representation may boost patient outcomes, by spreading awareness of the appearance of skin conditions on darker skin tones and encouraging patients to promptly seek care. The current state of SoC representation reflects health disparities, and we hope to encourage diversity not only in the literature but across social media platforms.

**Figure 1:**
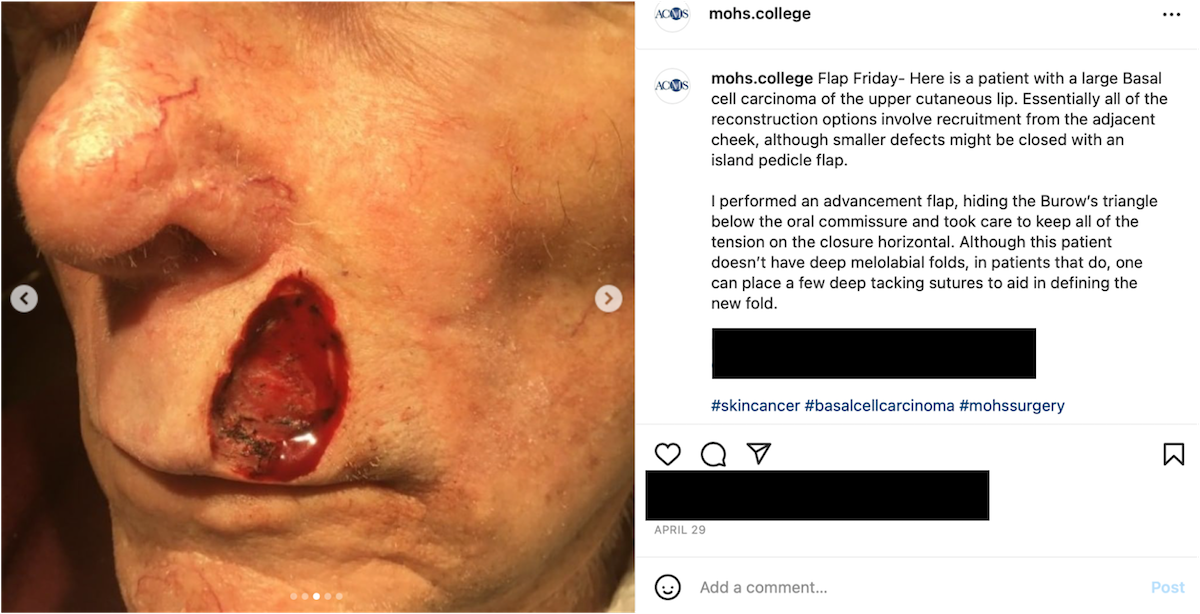
Example of a highly viewed American College of Mohs Surgery “Flap Friday” case on Instagram (@mohs.college), posted on April 29, 2022, showcasing Mohs patient photos and the clinical approach (accessed September 20, 2022).

**Figure 2:**
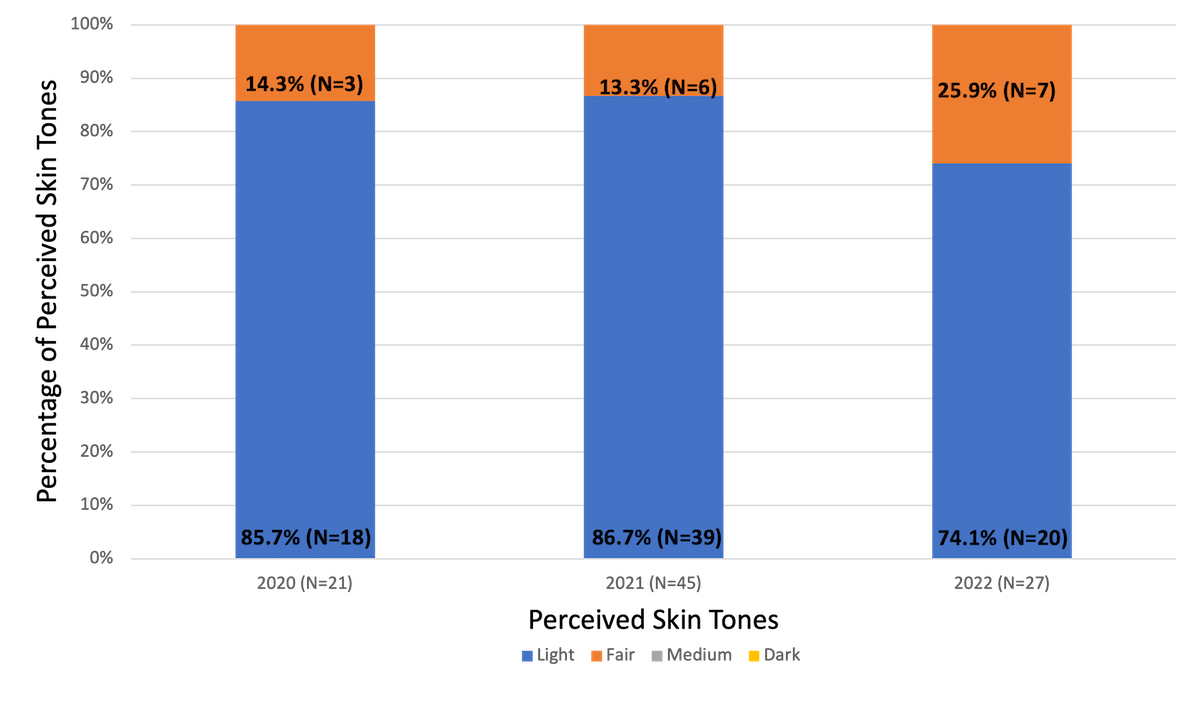
Percentages of perceived light, fair, medium, and dark skin tones depicted by weekly American College of Mohs Surgery “Flap Friday” cases on Instagram (@mohs.college) from 2020 to 2022.

## Abbreviations

ACMS: American College of Mohs Surgery
SoC: skin of color
